# Supplementary material for: Changes in operation of postural networks in rabbits with postural functions recovered after lateral hemisection of the spinal cord
Source: J Physiol. 2022 Dec 18;601(2):307–34. doi: 10.1113/JP283458 (PMC9840688; doi:10.1113/JP283458)
Supplement: Supplementary file 1 — Statistical Summary Document [file TJP-601-307-s001.docx]

**Manuscript Title:** Changes in operation of postural networks in rabbits with postural functions recovered after lateral hemisection of the spinal cord.

**Authors:** Pavel V. Zelenin, Vladimir F. Lyalka, and Tatiana G. Deliagina

**Animal model used, if applicable:** adult male New Zealand rabbits (Oryctolagus cuniculus)

- **Underlying hypothesis:** This study tests the hypothesis that activity of individual neurons in spinal postural network changes during recovery of the postural functions after the lateral hemisection of the spinal cord.

**Definitions of ‘n’:**

Question 1: N = number of animals

Questions 2-4: N = number of animals.

Questions 5-16: N = number of animals, n = number of neurons.

Questions 17: N = number of conditions.

Questions 18-29: N = number of animals, n = number of neurons.

**Statistical summary table:**

| Experimental question number* | Finding/ conclusion | Experimental location/ variable  e.g. cortex vs cerebellum or genotype | Mean value  (or other summary statistic) | SD | n (value) | P** | Units | Data comparisons  e.g. WT vs KO | Statistical test | Any other variable  e.g. subjects’ age or sex | Figure/table in which data are presented | Comments  e.g. observation |
| --- | --- | --- | --- | --- | --- | --- | --- | --- | --- | --- | --- | --- |
| 1. Gain of postural corrections? | No significant difference | R-rabbits | 0.081 | 0.045 | N = 8 | 0.526 | cm / degree | R-rabbits vs intact rabbits | Welch’s  t-test | Rabbits before decerebration | Fig. 2B |  |
|  |  | intact rabbits (control) | 0.069 | 0.022 | N = 9 |  |  |  |  |  |  |  |
| 2. Percentage of correct EMG responses? | Lower in  R-rabbits | ipsi-LHS  in R-rabbits | 77.0 | 29.2 | N = 8 | **9 x 10^-4^** | % of tilt cycles | R-rabbits  vs  intact rabbits | Welch’s  t-test | Rabbits before decerebration | Fig. 2A |  |
|  |  | in intact rabbits (control) | 100.0 | 0.0 | N = 9 |  |  |  |  |  |  |  |
|  |  | co-LHS  in R-rabbits | 79.2 | 34.1 | N = 8 | **0.033** |  |  |  |  |  |  |
|  |  | intact rabbits (control) | 100.0 | 0.0 | N = 9 |  |  |  |  |  |  |  |
| 3. Percentage of correct EMG responses? | On damaged side  higher in  R-rabbits | ipsi-LHS  in R-rabbits | 74.3 | 39.7 | N = 8 | **0.022** | % of tilt cycles | R-rabbits  vs  rabbits after acute LHS | Welch’s  t-test | Decerebrated preparations | Fig. 2C |  |
|  |  | ipsi-LHS  after acute LHS | 24.5 | 37.5 | N = 7 |  |  |  |  |  |  |  |
|  | On intact side  lower in  R-rabbits | co-LHS  in R-rabbits | 42.4 | 48.5 | N = 8 | **0.001** |  |  |  |  |  |  |
|  |  | co-LHS  after acute LHS | 85.3 | 22.6 | N = 7 |  |  |  |  |  |  |  |
| 4. Percentage of correct EMG responses? | No significant difference | Responses to  whole platform tilts  ipsi-LHS | 74.3 | 39.7 | N = 8 | 0.957 | % of tilt cycles | Responses to whole platform tilts  vs  responses to tilts of ipsilateral platform | Welch’s  t-test | Decerebrated R-rabbits | Fig. 2C Fig. 2D |  |
|  |  | Responses to  ipsilateral platform tilts  ipsi-LHS | 73.2 | 34.5 | N = 8 |  |  |  |  |  |  |  |
|  |  | Responses to  whole platform tilts  co-LHS | 42.4 | 48.5 | N = 8 | 0.852 |  |  |  |  |  |  |
|  |  | Responses to  ipsilateral platform tilts  co-LHS | 45.7 | 38.0 | N = 8 |  |  |  |  |  |  |  |
|  | Higher for whole platform tilts | Responses to whole platform tilts  ipsi-LHS | 74.3 | 39.7 | N = 8 | **0.0013** |  | Responses to whole platform tilts  vs  responses to tilts of contralateral platform |  |  | Fig. 2C  Fig. 2E |  |
|  |  | Responses to contralateral platform tilts  ipsi-LHS | 4.2 | 11.8 | N = 8 |  |  |  |  |  |  |  |
|  |  | Responses to whole platform tilts  co-LHS | 42.4 | 48.5 | N = 8 | **0.008** |  |  |  |  |  |  |
|  |  | Responses to contralateral platform tilts  co-LHS | 0.0 | 0.0 | N = 8 |  |  |  |  |  |  |  |
| 5. Percentage of F-neurons? | Lower in R-rabbits | ipsi-LHS  in R-rabbits | 38.4 |  | N = 8,  n = 185 | **0.023** | % of neurons | R-rabbits  vs  intact rabbits | χ^2^  test | Decerebrated preparations | Fig. 3C |  |
|  |  | intact rabbits (control) | 48.6 |  | N = 15,  n = 360 |  |  |  |  |  |  |  |
|  |  | co-LHS  in R-rabbits | 39.7 |  | N = 8,  n = 194 | **0.044** |  |  |  |  |  |  |
|  |  | intact rabbits (control) | 48.6 |  | N = 15,  n = 360 |  |  |  |  |  |  |  |
|  | No significant difference | ipsi-LHS  in R-rabbits | 38.4 |  | N = 8,  n = 185 | 0.408 |  | R-rabbits  vs  rabbits after acute LHS |  |  |  |  |
|  |  | ipsi-LHS  after acute LHS | 34.4 |  | N = 7,  n = 218 |  |  |  |  |  |  |  |
|  |  | co-LHS  in R-rabbits | 39.7 |  | N = 8,  n = 194 | 0.267 |  |  |  |  |  |  |
|  |  | co-LHS  after acute LHS | 44.9 |  | N = 7,  n = 272 |  |  |  |  |  |  |  |
| 6. Percentage of E-neurons? | No significant difference | ipsi-LHS  in R-rabbits | 30.3 |  | N = 8,  n = 185 | 0.430 | % of neurons | R-rabbits  vs  intact rabbits | χ^2^  test | Decerebrated preparations | Fig. 3C |  |
|  |  | intact rabbits (control) | 33.6 |  | N = 15,  n = 360 |  |  |  |  |  |  |  |
|  |  | co-LHS  in R-rabbits | 27.3 |  | N = 8,  n = 194 | 0.128 |  |  |  |  |  |  |
|  |  | intact rabbits (control) | 33.6 |  | N = 15,  n = 360 |  |  |  |  |  |  |  |
|  |  | ipsi-LHS  in R-rabbits | 30.3 |  | N = 8,  n = 185 | 0.174 |  | R-rabbits  vs  rabbits after acute LHS |  |  |  |  |
|  |  | ipsi-LHS  after acute LHS | 36.7 |  | N = 7,  n = 218 |  |  |  |  |  |  |  |
|  |  | co-LHS  in R-rabbits | 27.3 |  | N = 8,  n = 194 | 0.279 |  |  |  |  |  |  |
|  |  | co-LHS  after acute LHS | 32.0 |  | N = 7,  n = 272 |  |  |  |  |  |  |  |
| 7. Percentage of  NM-neurons? | Higher in R-rabbits | ipsi-LHS  in R-rabbits | 31.4 |  | N = 8,  n = 185 | **3 x 10^-4^** | % of neurons | R-rabbits  vs  intact rabbits | χ^2^  test | Decerebrated preparations | Fig. 3C |  |
|  |  | intact rabbits (control) | 17.8 |  | N = 15,  n = 360 |  |  |  |  |  |  |  |
|  |  | co-LHS  in R-rabbits | 33.0 |  | N = 8,  n = 194 | **5 x 10^-5^** |  |  |  |  |  |  |
|  |  | intact rabbits (control) | 17.8 |  | N = 15,  n = 360 |  |  |  |  |  |  |  |
|  | No significant difference | ipsi-LHS  in R-rabbits | 31.4 |  | N = 8,  n = 185 | 0.593 |  | R-rabbits  vs  rabbits after acute LHS |  |  |  |  |
|  |  | ipsi-LHS  after acute LHS | 28.9 |  | N = 7,  n = 218 |  |  |  |  |  |  |  |
|  | Higher  in R-rabbits | co-LHS  in R-rabbits | 33.0 |  | N = 8,  n = 194 | **0.019** |  |  |  |  |  |  |
|  |  | co-LHS  after acute LHS | 23.2 |  | N = 7,  n = 272 |  |  |  |  |  |  |  |
| 8. Mean frequency  in NM-neurons? | No significant difference | Zone 1  ipsi-LHS  in R-rabbits | 9.7 | 10.6 | N = 8,  n = 10 | 0.847 | Hz | R-rabbits  vs  intact rabbits | Welch’s  t-test | Decerebrated preparations | Fig. 3D |  |
|  |  | Zone 1  intact rabbits (control) | 10.6 | 10.7 | N = 15,  n = 14 |  |  |  |  |  |  |  |
|  |  | Zone 2  ipsi-LHS  in R-rabbits | 8.5 | 7.3 | N = 8,  n = 24 | 0.817 |  |  |  |  |  |  |
|  |  | Zone 2  intact rabbits (control) | 9.0 | 7.0 | N = 15,  n = 27 |  |  |  |  |  |  |  |
|  |  | Zone 3  ipsi-LHS  in R-rabbits | 9.3 | 7.6 | N = 8,  n = 24 | 0.488 |  |  |  |  |  |  |
|  |  | Zone 3  intact rabbits (control) | 11.1 | 9.7 | N = 15,  n = 23 |  |  |  |  |  |  |  |
|  | No significant difference | Zone 1  co-LHS  in R-rabbits | 15.4 | 13.4 | N = 8,  n = 9 | 0.373 |  |  |  |  |  |  |
|  |  | Zone 1  intact rabbits (control) | 10.6 | 10.7 | N = 15,  n = 14 |  |  |  |  |  |  |  |
|  |  | Zone 2  co-LHS  in R-rabbits | 8.7 | 8.6 | N = 8,  n = 25 | 0.912 |  |  |  |  |  |  |
|  |  | Zone 2  intact rabbits (control) | 9.0 | 7.0 | N = 15,  n = 27 |  |  |  |  |  |  |  |
|  |  | Zone 3  co-LHS  in R-rabbits | 17.2 | 18.4 | N = 8,  n = 30 | 0.122 |  |  |  |  |  |  |
|  |  | Zone 3  intact rabbits (control) | 11.1 | 9.7 | N = 15,  n = 23 |  |  |  |  |  |  |  |
| 9. Mean frequency  in F-neurons? | Lower in R-rabbits | Zone 1  ipsi-LHS  in R-rabbits | 8.3 | 5.2 | N = 8,  n = 22 | **0.007** | Hz | R-rabbits  vs  intact rabbits | Welch’s  t-test | Decerebrated preparations | Fig. 4A |  |
|  |  | Zone 1  intact rabbits (control) | 12.3 | 7.2 | N = 20,  n = 62 |  |  |  |  |  |  |  |
|  | No significant difference | Zone 2  ipsi-LHS  in R-rabbits | 13.5 | 12.7 | N = 8,  n = 24 | 0.348 |  |  |  |  |  |  |
|  |  | Zone 2  intact rabbits (control) | 10.8 | 10.1 | N = 20,  n = 94 |  |  |  |  |  |  |  |
|  |  | Zone 3  ipsi-LHS  in R-rabbits | 12.6 | 10.2 | N = 8,  n = 25 | 0.125 |  |  |  |  |  |  |
|  |  | Zone 3  intact rabbits (control) | 9.1 | 7.4 | N = 20,  n = 93 |  |  |  |  |  |  |  |
|  | No significant difference | Zone 1  co-LHS  in R-rabbits | 11.8 | 12.7 | N = 8,  n = 25 | 0.874 |  |  |  |  |  |  |
|  |  | Zone 1  intact rabbits (control) | 12.3 | 7.2 | N = 20,  n = 62 |  |  |  |  |  |  |  |
|  |  | Zone 2  co-LHS  in R-rabbits | 8.0 | 8.2 | N = 8,  n = 26 | 0.152 |  |  |  |  |  |  |
|  |  | Zone 2  intact rabbits (control) | 10.8 | 10.1 | N = 20,  n = 94 |  |  |  |  |  |  |  |
|  |  | Zone 3  co-LHS  in R-rabbits | 11.6 | 7.9 | N = 8,  n = 26 | 0.172 |  |  |  |  |  |  |
|  |  | Zone 3  intact rabbits (control) | 9.1 | 7.4 | N = 20,  n = 93 |  |  |  |  |  |  |  |
| 10. Depth of modulation  in F-neurons? | No significant difference | Zone 1  ipsi-LHS  in R-rabbits | 13.2 | 8.3 | N = 8,  n = 22 | 0.100 | Hz | R-rabbits  vs  intact rabbits | Welch’s  t-test | Decerebrated preparations | Fig. 4B |  |
|  |  | Zone 1  intact rabbits (control) | 17.1 | 11.8 | N = 20,  n = 62 |  |  |  |  |  |  |  |
|  |  | Zone 2  ipsi-LHS  in R-rabbits | 10.9 | 9.4 | N = 8,  n = 24 | 0.675 |  |  |  |  |  |  |
|  |  | Zone 2  intact rabbits (control) | 11.8 | 11.6 | N = 20,  n = 94 |  |  |  |  |  |  |  |
|  |  | Zone 3  ipsi-LHS  in R-rabbits | 11.1 | 7.4 | N = 8,  n = 25 | 0.168 |  |  |  |  |  |  |
|  |  | Zone 3  intact rabbits (control) | 8.8 | 6.8 | N = 20,  n = 93 |  |  |  |  |  |  |  |
|  | No significant difference  Higher  in R-rabbits | Zone 1  co-LHS  in R-rabbits | 13.4 | 12.6 | N = 8,  n = 25 | 0.220 |  |  |  |  |  |  |
|  |  | Zone 1  intact rabbits (control) | 17.1 | 11.8 | N = 20,  n = 62 |  |  |  |  |  |  |  |
|  |  | Zone 2  co-LHS  in R-rabbits | 9.3 | 12.0 | N = 8,  n = 26 | 0.348 |  |  |  |  |  |  |
|  |  | Zone 2  intact rabbits (control) | 11.8 | 11.6 | N = 20,  n = 94 |  |  |  |  |  |  |  |
|  |  | Zone 3  co-LHS  in R-rabbits | 12.5 | 8.8 | N = 8,  n = 26 | 0.051 |  |  |  |  |  |  |
|  |  | Zone 3  intact rabbits (control) | 8.8 | 6.8 | N = 20,  n = 93 |  |  |  |  |  |  |  |
| 11. Burst frequency  in F-neurons? | Lower  in R-rabbits | Zone 1  ipsi-LHS  in R-rabbits | 14.9 | 9.0 | N = 8,  n = 22 | **0.020** | Hz | R-rabbits  vs  intact rabbits | Welch’s  t-test | Decerebrated preparations | Fig. 4C |  |
|  |  | Zone 1  intact rabbits (control) | 20.8 | 12.4 | N = 20,  n = 62 |  |  |  |  |  |  |  |
|  | No significant difference | Zone 2  ipsi-LHS  in R-rabbits | 18.9 | 15.7 | N = 8,  n = 24 | 0.544 |  |  |  |  |  |  |
|  |  | Zone 2  intact rabbits (control) | 16.7 | 14.8 | N = 20,  n = 94 |  |  |  |  |  |  |  |
|  |  | Zone 3  ipsi-LHS  in R-rabbits | 18.1 | 11.8 | N = 8,  n = 25 | 0.083 |  |  |  |  |  |  |
|  |  | Zone 3  intact rabbits (control) | 13.5 | 10.0 | N = 20,  n = 93 |  |  |  |  |  |  |  |
|  | No significant difference | Zone 1  co-LHS  in R-rabbits | 18.6 | 18.4 | N = 8,  n = 25 | 0.575 |  |  |  |  |  |  |
|  |  | Zone 1  intact rabbits (control) | 20.8 | 12.4 | N = 20,  n = 62 |  |  |  |  |  |  |  |
|  |  | Zone 2  co-LHS  in R-rabbits | 12.7 | 13.1 | N = 8,  n = 26 | 0.184 |  |  |  |  |  |  |
|  |  | Zone 2  intact rabbits (control) | 16.7 | 14.8 | N = 20,  n = 94 |  |  |  |  |  |  |  |
|  |  | Zone 3  co-LHS  in R-rabbits | 17.9 | 11.3 | N = 8,  n = 26 | 0.079 |  |  |  |  |  |  |
|  |  | Zone 3  intact rabbits (control) | 13.5 | 10.0 | N = 20,  n = 93 |  |  |  |  |  |  |  |
| 12. Interburst frequency  in F-neurons? | Lower  in R-rabbits | Zone 1  ipsi-LHS  in R-rabbits | 1.7 | 2.7 | N = 8,  n = 22 | **0.015** | Hz | R-rabbits  vs  intact rabbits | Welch’s  t-test | Decerebrated preparations | Fig. 4D |  |
|  |  | Zone 1  intact rabbits (control) | 3.7 | 4.7 | N = 20,  n = 62 |  |  |  |  |  |  |  |
|  | No significant difference | Zone 2  ipsi-LHS  in R-rabbits | 8.0 | 10.9 | N = 8,  n = 24 | 0.191 |  |  |  |  |  |  |
|  |  | Zone 2  intact rabbits (control) | 4.9 | 7.2 | N = 20,  n = 94 |  |  |  |  |  |  |  |
|  |  | Zone 3  ipsi-LHS  in R-rabbits | 7.1 | 9.9 | N = 8,  n = 25 | 0.275 |  |  |  |  |  |  |
|  |  | Zone 3  intact rabbits (control) | 4.8 | 5.9 | N = 20,  n = 93 |  |  |  |  |  |  |  |
|  | No significant difference | Zone 1  co-LHS  in R-rabbits | 5.1 | 8.0 | N = 8,  n = 25 | 0.418 |  |  |  |  |  |  |
|  |  | Zone 1  intact rabbits (control) | 3.7 | 4.7 | N = 20,  n = 62 |  |  |  |  |  |  |  |
|  |  | Zone 2  co-LHS  in R-rabbits | 3.4 | 5.9 | N = 8,  n = 26 | 0.271 |  |  |  |  |  |  |
|  |  | Zone 2  intact rabbits (control) | 4.9 | 7.2 | N = 20,  n = 94 |  |  |  |  |  |  |  |
|  |  | Zone 3  co-LHS  in R-rabbits | 5.2 | 6.3 | N = 8,  n = 26 | 0.778 |  |  |  |  |  |  |
|  |  | Zone 3  intact rabbits (control) | 4.8 | 5.9 | N = 20,  n = 93 |  |  |  |  |  |  |  |
| 13. Mean frequency  in E-neurons? | No significant difference | Zone 1  ipsi-LHS  in R-rabbits | 10.3 | 9.6 | N = 8,  n = 14 | 0.953 | Hz | R-rabbits  vs  intact rabbits | Welch’s  t-test | Decerebrated preparations | Fig. 5A |  |
|  |  | Zone 1  intact rabbits (control) | 10.4 | 5.9 | N = 20,  n = 49 |  |  |  |  |  |  |  |
|  |  | Zone 2  ipsi-LHS  in R-rabbits | 13.1 | 13.2 | N = 8,  n = 18 | 0.302 |  |  |  |  |  |  |
|  |  | Zone 2  intact rabbits (control) | 9.6 | 7.6 | N = 20,  n = 63 |  |  |  |  |  |  |  |
|  |  | Zone 3  ipsi-LHS  in R-rabbits | 9.1 | 7.8 | N = 8,  n = 24 | 0.796 |  |  |  |  |  |  |
|  |  | Zone 3  intact rabbits (control) | 9.6 | 6.9 | N = 20,  n = 74 |  |  |  |  |  |  |  |
|  | Lower  in R-rabbits | Zone 1  co-LHS  in R-rabbits | 6.6 | 3.9 | N = 8,  n = 7 | **0.046** |  |  |  |  |  |  |
|  |  | Zone 1  intact rabbits (control) | 10.4 | 5.9 | N = 20,  n = 49 |  |  |  |  |  |  |  |
|  | No significant difference | Zone 2  co-LHS  in R-rabbits | 12.7 | 13.8 | N = 8,  n = 22 | 0.324 |  |  |  |  |  |  |
|  |  | Zone 2  intact rabbits (control) | 9.6 | 7.6 | N = 20,  n = 63 |  |  |  |  |  |  |  |
|  |  | Zone 3  co-LHS  in R-rabbits | 13.0 | 12.0 | N = 8,  n = 24 | 0.188 |  |  |  |  |  |  |
|  |  | Zone 3  intact rabbits (control) | 9.6 | 6.9 | N = 20,  n = 74 |  |  |  |  |  |  |  |
| 14. Depth of modulation  in E-neurons? | No significant difference | Zone 1  ipsi-LHS  in R-rabbits | 12.1 | 11.5 | N = 8,  n = 14 | 0.450 | Hz | R-rabbits  vs  intact rabbits | Welch’s  t-test | Decerebrated preparations | Fig. 5B |  |
|  |  | Zone 1  intact rabbits (control) | 14.7 | 9.1 | N = 20,  n = 49 |  |  |  |  |  |  |  |
|  |  | Zone 2  ipsi-LHS  in R-rabbits | 12.2 | 10.7 | N = 8,  n = 18 | 0.619 |  |  |  |  |  |  |
|  |  | Zone 2  intact rabbits (control) | 10.7 | 12.7 | N = 20,  n = 63 |  |  |  |  |  |  |  |
|  |  | Zone 3  ipsi-LHS  in R-rabbits | 8.4 | 5.3 | N = 8,  n = 24 | 0.612 |  |  |  |  |  |  |
|  |  | Zone 3  intact rabbits (control) | 7.8 | 5.5 | N = 20,  n = 74 |  |  |  |  |  |  |  |
|  | Lower  in R-rabbits | Zone 1  co-LHS  in R-rabbits | 8.5 | 5.6 | N = 8,  n = 7 | **0.028** |  |  |  |  |  |  |
|  |  | Zone 1  intact rabbits (control) | 14.7 | 9.1 | N = 20,  n = 49 |  |  |  |  |  |  |  |
|  | No significant difference | Zone 2  co-LHS  in R-rabbits | 10.9 | 7.1 | N = 8,  n = 22 | 0.920 |  |  |  |  |  |  |
|  |  | Zone 2  intact rabbits (control) | 10.7 | 12.7 | N = 20,  n = 63 |  |  |  |  |  |  |  |
|  | Higher  in R-rabbits | Zone 3  co-LHS  in R-rabbits | 13.6 | 11.9 | N = 8,  n = 24 | **0.030** |  |  |  |  |  |  |
|  |  | Zone 3  intact rabbits (control) | 7.8 | 5.5 | N = 20,  n = 74 |  |  |  |  |  |  |  |
| 15. Burst frequency  in E-neurons? | No significant difference | Zone 1  ipsi-LHS  in R-rabbits | 16.3 | 13.9 | N = 8,  n = 14 | 0.718 | Hz | R-rabbits  vs  intact rabbits | Welch’s  t-test | Decerebrated preparations | Fig. 5C |  |
|  |  | Zone 1  intact rabbits (control) | 17.8 | 9.4 | N = 20,  n = 49 |  |  |  |  |  |  |  |
|  |  | Zone 2  ipsi-LHS  in R-rabbits | 19.2 | 17.4 | N = 8,  n = 18 | 0.351 |  |  |  |  |  |  |
|  |  | Zone 2  intact rabbits (control) | 15.0 | 13.1 | N = 20,  n = 63 |  |  |  |  |  |  |  |
|  |  | Zone 3  ipsi-LHS  in R-rabbits | 13.3 | 9.8 | N = 8,  n = 24 | 0.949 |  |  |  |  |  |  |
|  |  | Zone 3  intact rabbits (control) | 13.5 | 8.6 | N = 20,  n = 74 |  |  |  |  |  |  |  |
|  | Lower  in R-rabbits | Zone 1  co-LHS  in R-rabbits | 10.8 | 6.3 | N = 8,  n = 7 | **0.029** |  |  |  |  |  |  |
|  |  | Zone 1  intact rabbits (control) | 17.8 | 9.4 | N = 20,  n = 49 |  |  |  |  |  |  |  |
|  | No significant difference | Zone 2  co-LHS  in R-rabbits | 18.2 | 16.2 | N = 8,  n = 22 | 0.406 |  |  |  |  |  |  |
|  |  | Zone 2  intact rabbits (control) | 15.0 | 13.1 | N = 20,  n = 63 |  |  |  |  |  |  |  |
|  |  | Zone 3  co-LHS  in R-rabbits | 19.8 | 17.3 | N = 8,  n = 24 | 0.095 |  |  |  |  |  |  |
|  |  | Zone 3  intact rabbits (control) | 13.5 | 8.6 | N = 20,  n = 74 |  |  |  |  |  |  |  |
| 16. Interburst frequency  in E-neurons? | No significant difference | Zone 1  ipsi-LHS  in R-rabbits | 4.2 | 9.6 | N = 8,  n = 14 | 0.600 | Hz | R-rabbits  vs  intact rabbits | Welch’s  t-test | Decerebrated preparations | Fig. 5D |  |
|  |  | Zone 1  intact rabbits (control) | 3.1 | 4.8 | N = 20,  n = 49 |  |  |  |  |  |  |  |
|  |  | Zone 2  ipsi-LHS  in R-rabbits | 7.0 | 10.2 | N = 8,  n = 18 | 0.290 |  |  |  |  |  |  |
|  |  | Zone 2  intact rabbits (control) | 4.3 | 4.8 | N = 20,  n = 63 |  |  |  |  |  |  |  |
|  |  | Zone 3  ipsi-LHS  in R-rabbits | 4.9 | 6.4 | N = 8,  n = 24 | 0.600 |  |  |  |  |  |  |
|  |  | Zone 3  intact rabbits (control) | 5.6 | 6.0 | N = 20,  n = 74 |  |  |  |  |  |  |  |
|  | No significant difference | Zone 1  co-LHS  in R-rabbits | 2.3 | 2.3 | N = 8,  n = 7 | 0.524 |  |  |  |  |  |  |
|  |  | Zone 1  intact rabbits (control) | 3.1 | 4.8 | N = 20,  n = 49 |  |  |  |  |  |  |  |
|  |  | Zone 2  co-LHS  in R-rabbits | 7.2 | 12.0 | N = 8,  n = 22 | 0.266 |  |  |  |  |  |  |
|  |  | Zone 2  intact rabbits (control) | 4.3 | 4.8 | N = 20,  n = 63 |  |  |  |  |  |  |  |
|  |  | Zone 3  co-LHS  in R-rabbits | 6.3 | 7.7 | N = 8,  n = 24 | 0.726 |  |  |  |  |  |  |
|  |  | Zone 3  intact rabbits (control) | 5.6 | 6.0 | N = 20,  n = 74 |  |  |  |  |  |  |  |
| 17. Correlation:  difference of correct and incorrect EMG responses  vs  difference of F and E responses | Positive correlation | Zones 2 and 3 | 0.746 | 0.019 | N = 5 | **0.001** |  |  | Ordinary least squares | Decerebrated preparations | Fig. 7A | R^2^=  0.99 |
|  | No significant correlation | Zone 1 | -0.506 | 0.237 |  | 0.273 |  |  |  |  | Fig. 7B | R^2^=  0.23 |
| 18. Percentage  of Type1  F- neurons? | Higher for R-rabbits | ipsi-LHS  in R-rabbits | 79.6 |  | N = 8,  n = 54 | **2 x 10^-6^** | % of neurons | R-rabbits  vs  intact rabbits (control) | χ^2^  test | Decerebrated R-rabbits | Fig. 8A |  |
|  |  | intact rabbits (control) | 42.9 |  | N = 20,  n = 175 |  |  |  |  |  |  |  |
|  |  | co-LHS  in R-rabbits | 78.7 |  | N = 8,  n = 61 | **10^-6^** |  |  |  |  |  |  |
|  |  | intact rabbits (control) | 42.9 |  | N = 20,  n = 175 |  |  |  |  |  |  |  |
|  | No significant difference | ipsi-LHS  in R-rabbits | 79.6 |  | N = 8,  n = 54 | 0.383 |  | R-rabbits  vs  rabbits after acute LHS |  |  |  |  |
|  |  | ipsi-LHS  after acute LHS | 72.3 |  | N = 7,  n = 70 |  |  |  |  |  |  |  |
|  |  | co-LHS  in R-rabbits | 78.7 |  | N = 8,  n = 61 | 0.878 |  |  |  |  |  |  |
|  |  | co-LHS  after acute LHS | 77.7 |  | N = 7,  n = 112 |  |  |  |  |  |  |  |
| 19. Percentage  of Type1  E- neurons? | Higher for R-rabbits | ipsi-LHS  in R-rabbits | 76.1 |  | N = 8,  n = 46 | **2 x 10^-7^** | % of neurons | R-rabbits  vs  intact rabbits (control) | χ^2^  test | Decerebrated R-rabbits | Fig. 9A |  |
|  |  | intact rabbits (control) | 31.8 |  | N = 20,  n = 132 |  |  |  |  |  |  |  |
|  |  | co-LHS  in R-rabbits | 63.4 |  | N = 8,  n = 41 | **3 x 10^-4^** |  |  |  |  |  |  |
|  |  | intact rabbits (control) | 31.8 |  | N = 20,  n = 132 |  |  |  |  |  |  |  |
|  | No significant difference | ipsi-LHS  in R-rabbits | 76.1 |  | N = 8,  n = 46 | 0.748 |  | R-rabbits  vs  rabbits after acute LHS |  |  |  |  |
|  |  | ipsi-LHS  after acute LHS | 73.0 |  | N = 7,  n = 74 |  |  |  |  |  |  |  |
|  |  | co-LHS  in R-rabbits | 63.4 |  | N = 8,  n = 41 | 0.148 |  |  |  |  |  |  |
|  |  | co-LHS  after acute LHS | 76.0 |  | N = 7,  n = 79 |  |  |  |  |  |  |  |
| 20. Depth of modulation for ipsi-platform tilts  in F-neurons? | No significant difference | Zone 1  ipsi-LHS  in R-rabbits | 14.3 | 9.1 | N = 8,  n = 15 | 0.398 | Hz | R-rabbits  vs  intact rabbits | Welch’s  t-test | Decerebrated preparations | Fig. 8B |  |
|  |  | Zone 1  intact rabbits (control) | 16.9 | 12.3 | N = 20,  n = 42 |  |  |  |  |  |  |  |
|  |  | Zone 2  ipsi-LHS  in R-rabbits | 9.5 | 10.8 | N = 8,  n = 19 | 0.754 |  |  |  |  |  |  |
|  |  | Zone 2  intact rabbits (control) | 10.4 | 11.4 | N = 20,  n = 62 |  |  |  |  |  |  |  |
|  |  | Zone 3  ipsi-LHS  in R-rabbits | 10.3 | 7.0 | N = 8,  n = 20 | 0.117 |  |  |  |  |  |  |
|  |  | Zone 3  intact rabbits (control) | 7.4 | 7.7 | N = 20,  n = 71 |  |  |  |  |  |  |  |
|  | Lower  in R-rabbits | Zone 1  co-LHS  in R-rabbits | 11.0 | 9.3 | N = 8,  n = 20 | **0.044** |  |  |  |  |  |  |
|  |  | Zone 1  intact rabbits (control) | 16.9 | 12.3 | N = 20,  n = 42 |  |  |  |  |  |  |  |
|  |  | Zone 2  co-LHS  in R-rabbits | 6.0 | 5.2 | N = 8,  n = 20 | **0.018** |  |  |  |  |  |  |
|  |  | Zone 2  intact rabbits (control) | 10.4 | 11.4 | N = 20,  n = 62 |  |  |  |  |  |  |  |
|  | No significant difference | Zone 3  co-LHS  in R-rabbits | 13.7 | 13.9 | N = 8,  n = 21 | 0.060 |  |  |  |  |  |  |
|  |  | Zone 3  intact rabbits (control) | 7.4 | 7.7 | N = 20,  n = 71 |  |  |  |  |  |  |  |
| 21. Depth of modulation for contra-platform tilts  in F-neurons? | Lower  in R-rabbits | Zone 1  ipsi-LHS  in R-rabbits | 0.45 | 0.65 | N = 8,  n = 15 | **0.002** | Hz | R-rabbits  vs  intact rabbits | Welch’s  t-test | Decerebrated preparations | Fig. 8C |  |
|  |  | Zone 1  intact rabbits (control) | 2.19 | 3.32 | N = 20,  n = 42 |  |  |  |  |  |  |  |
|  |  | Zone 2  ipsi-LHS  in R-rabbits | 1.01 | 1.22 | N = 8,  n = 19 | **0.015** |  |  |  |  |  |  |
|  |  | Zone 2  intact rabbits (control) | 2.81 | 5.29 | N = 20,  n = 62 |  |  |  |  |  |  |  |
|  |  | Zone 3  ipsi-LHS  in R-rabbits | 1.94 | 2.42 | N = 8,  n = 20 | **0.010** |  |  |  |  |  |  |
|  |  | Zone 3  intact rabbits (control) | 3.83 | 3.73 | N = 20,  n = 71 |  |  |  |  |  |  |  |
|  | No significant difference | Zone 1  co-LHS  in R-rabbits | 1.06 | 1.39 | N = 8,  n = 20 | 0.066 |  |  |  |  |  |  |
|  |  | Zone 1  intact rabbits (control) | 2.19 | 3.32 | N = 20,  n = 42 |  |  |  |  |  |  |  |
|  | Lower  in R-rabbits | Zone 2  co-LHS  in R-rabbits | 1.19 | 1.88 | N = 8,  n = 20 | **0.044** |  |  |  |  |  |  |
|  |  | Zone 2  intact rabbits (control) | 2.81 | 5.29 | N = 20,  n = 62 |  |  |  |  |  |  |  |
|  |  | Zone 3  co-LHS  in R-rabbits | 2.07 | 2.69 | N = 8,  n = 21 | **0.021** |  |  |  |  |  |  |
|  |  | Zone 3  intact rabbits (control) | 3.83 | 3.73 | N = 20,  n = 71 |  |  |  |  |  |  |  |
| 22. Depth of modulation for ipsi-platform tilts  in E-neurons? | No significant difference | Zone 1  ipsi-LHS  in R-rabbits | 11.2 | 11.5 | N = 8,  n = 13 | 0.852 | Hz | R-rabbits  vs  intact rabbits | Welch’s  t-test | Decerebrated preparations | Fig. 9B |  |
|  |  | Zone 1  intact rabbits (control) | 10.5 | 8.4 | N = 20,  n = 32 |  |  |  |  |  |  |  |
|  |  | Zone 2  ipsi-LHS  in R-rabbits | 11.0 | 12.0 | N = 8,  n = 13 | 0.467 |  |  |  |  |  |  |
|  |  | Zone 2  intact rabbits (control) | 8.2 | 10.0 | N = 20,  n = 45 |  |  |  |  |  |  |  |
|  |  | Zone 3  ipsi-LHS  in R-rabbits | 6.1 | 5.6 | N = 8,  n = 20 | 0.507 |  |  |  |  |  |  |
|  |  | Zone 3  intact rabbits (control) | 5.1 | 5.6 | N = 20,  n = 55 |  |  |  |  |  |  |  |
|  | No significant difference | Zone 1  co-LHS  in R-rabbits | 7.5 | 6.2 | N = 8,  n = 6 | 0.341 |  |  |  |  |  |  |
|  |  | Zone 1  intact rabbits (control) | 10.5 | 8.4 | N = 20,  n = 32 |  |  |  |  |  |  |  |
|  |  | Zone 2  co-LHS  in R-rabbits | 6.8 | 5.8 | N = 8,  n = 16 | 0.506 |  |  |  |  |  |  |
|  |  | Zone 2  intact rabbits (control) | 8.2 | 10.0 | N = 20,  n = 45 |  |  |  |  |  |  |  |
|  |  | Zone 3  co-LHS  in R-rabbits | 8.7 | 11.0 | N = 8,  n = 19 | 0.193 |  |  |  |  |  |  |
|  |  | Zone 3  intact rabbits (control) | 5.1 | 5.6 | N = 20,  n = 55 |  |  |  |  |  |  |  |
| 23. Depth of modulation for contra-platform tilts  in E-neurons? | No significant difference | Zone 1  ipsi-LHS  in R-rabbits | 2.49 | 3.89 | N = 8,  n = 13 | 0.081 | Hz | R-rabbits  vs  intact rabbits | Welch’s  t-test | Decerebrated preparations | Fig. 9C |  |
|  |  | Zone 1  intact rabbits (control) | 6.42 | 10.83 | N = 20,  n = 32 |  |  |  |  |  |  |  |
|  | Lower  in R-rabbits | Zone 2  ipsi-LHS  in R-rabbits | 1.21 | 1.80 | N = 8,  n = 13 | **0.033** |  |  |  |  |  |  |
|  |  | Zone 2  intact rabbits (control) | 5.46 | 12.55 | N = 20,  n = 45 |  |  |  |  |  |  |  |
|  |  | Zone 3  ipsi-LHS  in R-rabbits | 2.41 | 2.91 | N = 8,  n = 20 | **0.003** |  |  |  |  |  |  |
|  |  | Zone 3  intact rabbits (control) | 5.41 | 5.52 | N = 20,  n = 55 |  |  |  |  |  |  |  |
|  | Lower  in R-rabbits | Zone 1  co-LHS  in R-rabbits | 1.48 | 1.81 | N = 8,  n = 6 | **0.021** |  |  |  |  |  |  |
|  |  | Zone 1  intact rabbits (control) | 6.42 | 10.83 | N = 20,  n = 32 |  |  |  |  |  |  |  |
|  | No significant difference | Zone 2  co-LHS  in R-rabbits | 2.04 | 2.57 | N = 8,  n = 16 | 0.090 |  |  |  |  |  |  |
|  |  | Zone 2  intact rabbits (control) | 5.46 | 12.55 | N = 20,  n = 45 |  |  |  |  |  |  |  |
|  |  | Zone 3  co-LHS  in R-rabbits | 4.00 | 2.80 | N = 8,  n = 19 | 0.155 |  |  |  |  |  |  |
|  |  | Zone 3  intact rabbits (control) | 5.41 | 5.52 | N = 20,  n = 55 |  |  |  |  |  |  |  |
| 24. Percentage of neurons with RF? | Lower in R-rabbits | ipsi-LHS  in R-rabbits | 74.0 |  | N = 8,  n = 73 | **0.010** | % of neurons | R-rabbits  vs  intact rabbits | χ^2^  test | Decerebrated preparations | Fig. 10A |  |
|  |  | intact rabbits (control) | 86.4 |  | N = 20,  n = 286 |  |  |  |  |  |  |  |
|  |  | co-LHS  in R-rabbits | 68.9 |  | N = 8,  n = 74 | **4 x 10^-4^** |  |  |  |  |  |  |
|  |  | intact rabbits (control) | 86.4 |  | N = 20,  n = 286 |  |  |  |  |  |  |  |
|  | No significant difference | ipsi-LHS  in R-rabbits | 74.0 |  | N = 8,  n = 73 | 0.475 |  | R-rabbits  vs  rabbits after acute LHS |  |  |  |  |
|  |  | ipsi-LHS  after acute LHS | 69.3 |  | N = 20,  n = 140 |  |  |  |  |  |  |  |
|  |  | co-LHS  in R-rabbits | 68.9 |  | N = 8,  n = 74 | 0.057 |  |  |  |  |  |  |
|  |  | co-LHS  after acute LHS | 80.0 |  | N = 20,  n = 180 |  |  |  |  |  |  |  |
| 25. Percentage of neurons with multiple deep RF? | No significant difference | ipsi-LHS  in R-rabbits | 32.9 |  | N = 8,  n = 73 | 0.379 | % of neurons | R-rabbits  vs  intact rabbits | χ^2^  test | Decerebrated preparations | Fig. 10A |  |
|  |  | intact rabbits (control) | 38.5 |  | N = 20,  n = 286 |  |  |  |  |  |  |  |
|  |  | co-LHS  in R-rabbits | 31.2 |  | N = 8,  n = 74 | 0.241 |  |  |  |  |  |  |
|  |  | intact rabbits (control) | 38.5 |  | N = 20,  n = 286 |  |  |  |  |  |  |  |
|  | No significant difference | ipsi-LHS  in R-rabbits | 32.9 |  | N = 8,  n = 73 | 0.071 |  | R-rabbits  vs  rabbits after acute LHS |  |  |  |  |
|  |  | ipsi-LHS  after acute LHS | 45.7 |  | N = 20,  n = 140 |  |  |  |  |  |  |  |
|  | Lower in R-rabbits | co-LHS  in R-rabbits | 31.2 |  | N = 8,  n = 74 | **0.012** |  |  |  |  |  |  |
|  |  | co-LHS  after acute LHS | 48.3 |  | N = 20,  n = 180 |  |  |  |  |  |  |  |
| 26. Percentage of neurons with RF from 1 muscle? | Lower in R-rabbits | ipsi-LHS  in R-rabbits | 28.8 |  | N = 8,  n = 73 | **0.045** | % of neurons | R-rabbits  vs  intact rabbits | χ^2^  test | Decerebrated preparations | Fig. 10A |  |
|  |  | intact rabbits (control) | 41.6 |  | N = 20,  n = 286 |  |  |  |  |  |  |  |
|  |  | co-LHS  in R-rabbits | 25.7 |  | N = 8,  n = 74 | **0.012** |  |  |  |  |  |  |
|  |  | intact rabbits (control) | 41.6 |  | N = 20,  n = 286 |  |  |  |  |  |  |  |
|  | No significant difference | ipsi-LHS  in R-rabbits | 28.8 |  | N = 8,  n = 73 | 0.408 |  | R-rabbits  vs  rabbits after acute LHS |  |  |  |  |
|  |  | ipsi-LHS  after acute LHS | 23.4 |  | N = 20,  n = 140 |  |  |  |  |  |  |  |
|  |  | co-LHS  in R-rabbits | 25.7 |  | N = 8,  n = 74 | 0.545 |  |  |  |  |  |  |
|  |  | co-LHS  after acute LHS | 29.4 |  | N = 20,  n = 180 |  |  |  |  |  |  |  |
| 27. Percentage of neurons with RF from skin? | No significant difference | ipsi-LHS  in R-rabbits | 12.3 |  | N = 8,  n = 73 | 0.081 | % of neurons | R-rabbits  vs  intact rabbits | χ^2^  test | Decerebrated preparations | Fig. 10A |  |
|  |  | intact rabbits (control) | 6.3 |  | N = 20,  n = 286 |  |  |  |  |  |  |  |
|  |  | co-LHS  in R-rabbits | 12.2 |  | N = 8,  n = 74 | 0.088 |  |  |  |  |  |  |
|  |  | intact rabbits (control) | 6.3 |  | N = 20,  n = 286 |  |  |  |  |  |  |  |
| 28. Percentage of neurons  with response  that can be  explained  by RF? | Higher in R-rabbits | ipsi-LHS  in R-rabbits | 55.6 |  | N = 8,  n = 27 | **4 x 10^-4^** | % of neurons | R-rabbits  vs  intact rabbits | χ^2^  test | Decerebrated preparations | Fig. 10B |  |
|  |  | intact rabbits (control) | 23.2 |  | N = 20,  n = 211 |  |  |  |  |  |  |  |
|  |  | co-LHS  in R-rabbits | 12.2 |  | N = 8,  n = 21 | **2 x 10^-5^** |  |  |  |  |  |  |
|  |  | intact rabbits (control) | 6.3 |  | N = 20,  n = 211 |  |  |  |  |  |  |  |
| 29. Percentage of neurons  with response  that cannot be  explained  by RF? | Lower in R-rabbits | ipsi-LHS  in R-rabbits | 22.2 |  | N = 8,  n = 27 | **0.012** | % of neurons | R-rabbits  vs  intact rabbits | χ^2^  test | Decerebrated preparations | Fig. 10B |  |
|  |  | intact rabbits (control) | 47.9 |  | N = 20,  n = 211 |  |  |  |  |  |  |  |
|  |  | co-LHS  in R-rabbits | 9.5 |  | N = 8,  n = 21 | **7 x 10^-4^** |  |  |  |  |  |  |
|  |  | intact rabbits (control) | 47.9 |  | N = 20,  n = 211 |  |  |  |  |  |  |  |
